# Clinicoradiological Findings of Benign Osteopetrosis: Report of Two New Cases

**DOI:** 10.5681/joddd.2012.031

**Published:** 2012-11-12

**Authors:** Elahe Tohidi, Ali Bagherpour

**Affiliations:** ^1^Assistant Professor, Department of Oral Radiology, Faculty of Dentistry, Kerman University of Medical Sciences, Kerman, Iran; ^2^Assistant Professor, Department of Oral Radiology, Faculty of Dentistry and Dental Research Center, Mashhad University of Medical Sciences, Mashhad, Iran

**Keywords:** Benign osteopetrosis, bone dysplasia, pathologic fractures

## Abstract

Osteopetrosis represents a heterogeneous group of rare, hereditary bone dysplasias that share the hallmark of increased bone density caused by osteoclast dysfunction. It can manifest through a spectrum of symptoms and severity, from neonatal onset with life-threatening complications ("malignant" autosomal recessive osteopetrosis) to two more benign conditions with the incidental radiographic findings, principally affecting adults (autosomal dominant osteopetrosis type I and type II). We report 2 new cases of autosomal dominant osteopetrosis type II. Both subjects were short in stature. Multiple healed fractures of long bones, diminished marrow spaces and hypoplastic maxillary sinuses were remarkable findings. To our knowledge they are the first reported cases of autosomal dominant type II of this disease in Iran.

## Introduction

Osteopetrosis is a rare genetic disorder resulting from a reduction in bone resorption relative to bone formation, which leads to generalized increased bone mass. Imbalanced bone turnover is a consequence of inadequate osteoclastic bone resorption, despite normal osteoblastic function.^1,2^ The disease is characterized by thickening of the cortical region, a decrease in the size of bone marrow spaces and generalized bone sclerosis.

A German radiologist, Albers-Schönberg, first described this bone disorder in 1904 and Karshner in 1926 coined the term osteopetrosis (stony bone) for the disease.^3^ The estimated incidence of the disease is 1 in 100,000–500,000 of the population.^4^ The characteristic radiological findings are usually sufficient for diagnosis of the disease.^5^ Depending on the severity of clinical manifestations, age of onset and type of inheritance, osteopetrosis (OP) is divided into three types: severe infantile malignant autosomal recessive OP, intermediate autosomal recessive OP, and autosomal dominant OP (adult benign OP).^6,7^

The infantile variant is diagnosed within the first year of life and is often fatal in early childhood. The clinical characteristics include growth retardation, dysmorphic features, neural palsies due to narrowing of bony canals, recurrent infections because of defects in immune system, and hematologic problems resulting from bone marrow failure.^4,5^

Intermediate variant of OP is rare and is diagnosed usually in the first decade of life, although x-rays are normal at birth. Unlike malignant OP, hematologic disorders are rare in this variant. Most patients survive into adulthood; however, the prognosis is poor.^4,5^

Adult OP is the most common and mildest form of the disease. Many patients are asymptomatic and are diagnosed only when osteomyelitis, particularly in the mandible, occurs. Other symptoms include bone pain, recurrent fractures, back pain and degenerative arthritis. Bone marrow function is not compromised in this variant of OP.^4^

There are two recognized subtypes of autosomal dominant osteopetrosis (ADO) based on clinical and radiographic features: In type I (ADO I), skull sclerosis mainly affects the cranial vault, the spine does not show much sclerosis, and the fracture rate is low. However, patients in type II (ADO II) have more pronounced sclerosis of the cranial base, and higher fracture risk, but nerve compression is rare unlike ADO I.^4,5,8^

Due to rare reported cases of ADO type II in the literature;^8-10^ the aim of this paper is to report two new cases of this entity and to demonstrate clinicoradiological findings associated with ADO type II.

## Case report

### Case 1

A 42-year-old woman, short in stature, presented to the Department of Oral and Maxillofacial Surgery, Mashhad Faculty of Dentistry, with chief complaints of pain and pus discharge from the right lower jaw for three months. She had a history of fracture of the left lower limb. Intraoral examination revealed multiple remained roots and a soft tissue swelling with a purulent exudate in the right mandibular alveolar ridge. To evaluate the radiographic findings of the lesion, a panoramic radiograph was requested.

**Figure 1:**
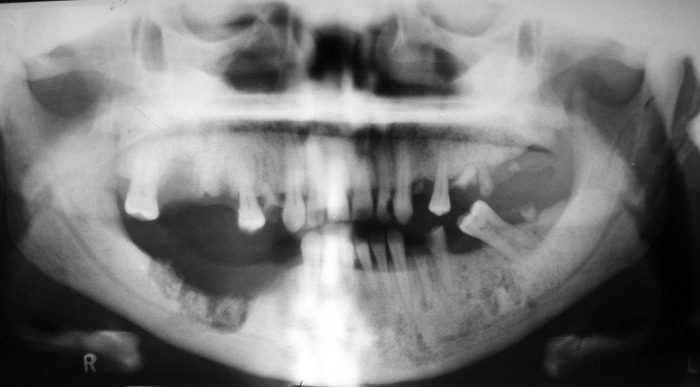
Panoramic radiograph of case 1, showing the intense maxillomandibular sclerosis and bony sequestrum on the right mandibular body.

Panoramic view demonstrated a generalized increase in radiodensity of the maxilla and mandible with abnormal trabecular pattern and diminished marrow spaces, multiple remained roots and decayed teeth. Maxillary sinuses were also hypoplastic (Figure 1).The presence of poorly healing extraction sockets in the right lower quadrant with evidence of bony sequestra circumscribed by an irregular radiolucency were noted, suggestive of a chronic osteomyelitis secondary to osteopetrosis. She was then referred to Shahid Kamyab Hospital, Mashhad University of Medical Sciences, for sequestrectomy and debridement of the necrotic bone.

**Figure 2:**
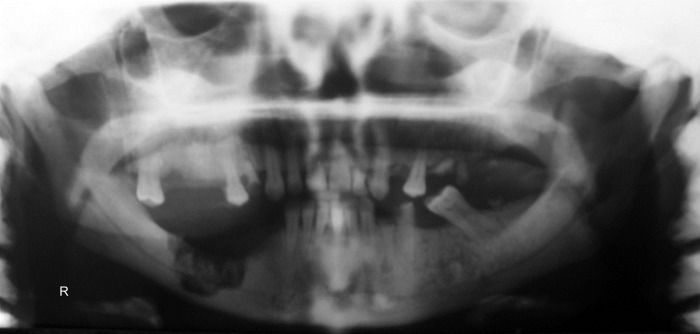
Panoramic radiograph of the first case three months later. Note the spread of osteomyelitis below the inferior alveolar canal and the presence of sequestra.

**Figure 3:**
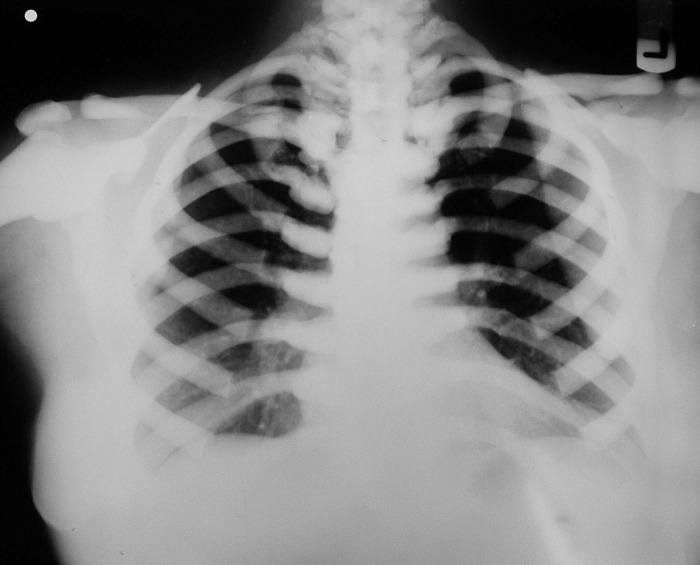
Posteroanterior chest x-ray of the first case showed a uniform increased bone density throughout the thoracic cage and both clavicular bones.

Unfortunately, a delay of three months to refer the patient for surgical treatment resulted in severe osteomyelitis in the region and spread of the infection into the submandibular space (Figure 2). Admission hematologic tests included red blood cell count, white blood cell count, platelet count, hemoglobin, and hematocrit; red blood cell indices were within the normal limits, as were erythrocyte sedimentation rate and coagulation profile. Posteroanterior (PA) chest x-ray revealed a uniform increase in bone density throughout the thoracic cage and both clavicular bones, with no abnormal finding in the chest parenchyma on either side (Figure 3).

Subsequently, the patient was administered intravenous antibiotics, after which sequestrectomy was performed. No evidence of recurrence was observed over three years of follow-up.

### Case 2

A 25-year-old man, with a height of 153 cm, referred to the Department of Oral Medicine, Mashhad Faculty of Dentistry, with a chief complaint of toothache in the right upper jaw. Intraoral examination showed multiple carious teeth in both jaws. The patient was referred to the Department of Oral and Maxillofacial Radiology for further investigation.

**Figure 4:**
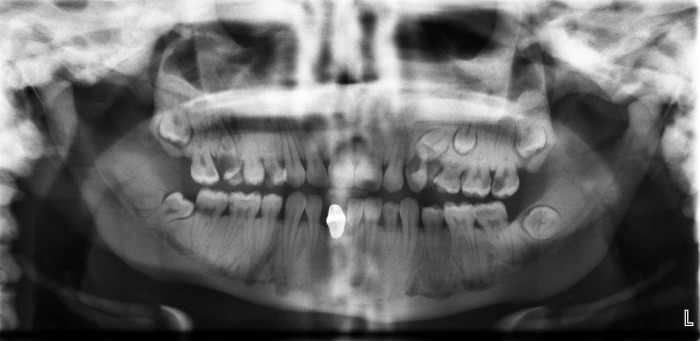
Panoramic radiograph of case 2 revealed generalized hyperdensity of the maxilla and mandible with multiple impacted teeth and carious lesions.

Panoramic radiograph revealed a general increase in bone density with absence of normal trabecular pattern involving both jaws, hypoplastic maxillary sinuses and multiple dental caries with pulpoperiapical pathoses. The impacted third molars and left upper quadrant premolars were seen with retained left maxillary deciduous molars and left mandibular lateral incisor. Bilateral missed second premolars of the mandible were also noted (Figure 4).

**Figure 5:**
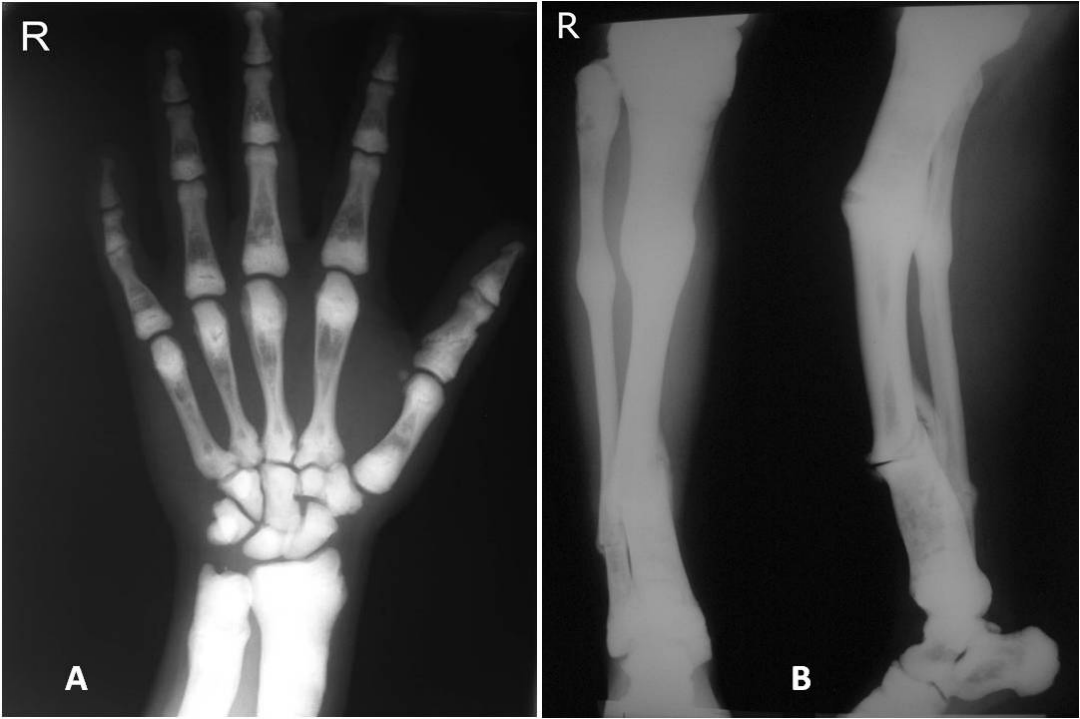
(A) PA hand-wrist radiograph revealed increased radiodensity. Note fracture of the proximal phalanx of the first finger and the presence of “bone-within-bone” appearance in metacarpal and phalangeal bones. (B) Anteroposterior and lateral views of the tibia and fibula demonstrated generalized osteosclerosis and multiple previous fractures.

Right hand posteroanterior (PA) radiograph showed hyperdense bones, particularly in the carpals, radius and ulna. Obliteration of cancellous bones, resulting in the loss of corticomedulary demarcation was observed. A previous fracture of the proximal phalanx of the first finger was also noted. The other radiographic finding of the hand x-ray was the presence of “bone-within-bone” appearance in metacarpal and phalangeal bones (Figure 5, A). In addition, the former anteroposterior (AP) and lateral views of the tibia and fibula demonstrated generalized osteosclerosis and multiple previous fractures (Figure 5, B).

The hemogram findings of the patient showed that his hemoglobin, hematocrit and RBC counts were lower than normal. An elevated erythrocyte sedimentation rate (ESR) was also noted. Serum calcium, phosphorous and alkaline phosphatase levels were within normal ranges.

According to the multiple maxillary pulpoperiapical lesions, he was referred to an oral and maxillofacial surgeon.

## Discussion

Bone turnover is a phenomenon that is highly regulated, resulting from the balance process of bone formation by osteoblasts and bone resorption by osteoclasts. Consequently, bone density is dependent on the relative activity of these two types of cells.^11^

Osteopetrosis is a family of bone diseases characterized by osteoclast failure. It is a rare genetic disorder, presenting with variable clinical features.^12^ In the most severe and fatal form, marrow spaces decrease, leading to hematological failure including anemia and pancytopenia.^4^ Even extramedullary hematopoiesis is unable to compensate for the reduction in medullary blood cell production with resultant hepatosplenomegaly, hypersplenism, and hemolysis.^13^ Neuropathies related to cranial nerve entrapment occurs, leading to visual and hearing deterioration.^14^ This type is the malignant variety, has an autosomal recessive inheritance, and usually results in death by the first decade of life.^3,13,15^ Autosomal recessive osteopetrosis also has intermediate severity, and the patients are often asymptomatic at birth but frequently exhibit fractures and psychomotor retardation by the end of their first decade of life. Most patients survive into adulthood but with significant disability.^3,4,5,13^

The most common variant of the disease is the benign adult autosomal dominant OP.^3^ The patients have normal life expectancy. This variant is the most heterogeneous in terms of severity and presents with a clinical spectrum which varies even within the same family.^16^

Anderson et al and others^17,18^ described two subtypes of benign autosomal dominant osteopetrosis (ADO) on the basis of radiological and clinical differences, including ADO Type I and ADO type II. Approximately 40% of patients with the adult form of osteopetrosis are symptom-free, regardless of type.^13,15^ Bone pain is common to both types.^13^ Bone marrow failure does not occur in benign osteopetrosis.^4^

In ADO Type I, the fracture rate is low because of the increased bone strength compared with normal. Radiographs reveal sclerosis of the skull, which mainly affects the cranial vault, with increased thickness of the vault. Cranial nerve compression is common in type I. In contrast to ADO II, the acid phosphatase levels are normal.^4,13^

The diagnosis of osteopetrosis is based on radiological and clinical features and these findings are sufficiently characteristic to make a definite diagnosis^3^ and there is no need to perform a genetic study to confirm the disease.^19^ Furthermore, in OP, biopsy must be avoided because of a marked infection risk.^12,19^ On the basis of radiological and clinical findings, our patients exhibited classical characteristics of the benign autosomal dominant osteopetrosis, although their family histories were unclear and it was not possible to examine their family members for determination of the exact hereditary pattern. Moreover, our patients appeared to match with the description of ADO type II, and the pathognomonic features of ADO type 2 such as “bone-within-bone’’ appearance in the vertebrae and extremities, and pathologic fractures were present in our cases. The second patient also exhibited mandibular osteomyelitis, which is the other classic manifestation of ADO type II.

The dental changes reported to be associated with OP include disturbance of tooth eruption due to impaired alveolar bone resorption of osteoclasts, hypodontia, malformed teeth, multiple caries, enamel dysplasia, abnormal pulp chambers, and hypercementosis.^3,7^ The increased bone density, initially presented as thickening of lamina dura, obscures the roots.^3,15^

Multiple dental caries, pulpoperiapical lesions, hypoplastic maxillary sinuses and the loss of contrast between the inferior cortical border and the cancellous portion of the bone were seen in both of our cases, consistent with those reported by several authors.^3,7,15,20^ Missing teeth, multiple tooth impactions, and retained deciduous tooth were observed in case 2.

Poor blood supply to the bone due to the bone marrow obliteration predisposes it to infection, as with our first patient. Osteomyelitis is generally caused by tooth extraction, pulpal necrosis, or periodontal infection.^12,13^ In case 1, osteomyelitis was the result of multiple dental extractions, which had been performed without any radiographic examination and regardless of underlying systemic condition.

The differential diagnosis that can be considered includes other sclerosing bone dysplasias, such as pyknodysostosis, craniometaphyseal dysplasia, diaphyseal dysplasia, melorheostosis, osteopoikilosis, and osteopathia striata. Fluoride poisoning and secondary hyperparathyroidism from renal osteodystrophy also may produce a diffuse ostesclerosis.^3,15^

Bone marrow function in benign OP is not compromised and the hematological findings are often normal. However, when osteoarthritis or osteomyelitis is present, the hematologic findings may become complicated because of the anemia of chronic disease. Calcium, phosphorus, and alkaline phosphatase levels in the benign form are usually within normal limits.^3,4^

In our first case, normal levels in blood test indicated a mild disease or a compensatory extramedullary erythropoietic function. However, the medullary obliteration was the result of reduced vascular supply to the bone, leading to osteomyelitis. The hemogram of the second case revealed a normocytic normochromic anemia, but the calcium-phosphorus metabolism and alkaline phosphatase level were normal. He had an increased erythrocyte sedimentation rate (35 mm/first hour), which could be due to his dental infection.

For patients with benign OP, the best treatment appears to be preventive management; therefore, they should be encouraged to maintain good dental care. Moreover, teeth should be endodontically treated, if possible, rather than extracted, due to the increased risk of infection. If osteomyelitis occurs, surgical intervention must be considered because antibiotics do not reach the compromised region.^19^ Treatment is still challenging and surgical procedures may vary from curettage to full resection.^5,19^ Nevertheless, in the review of literature, there is a tendency to treat these patients in the most conservative approach because of the diminished vascularity of the bone and the patient’s systemic condition.^19^ However, there are few reports of successful treatment of osteomyelitis secondary to OP and many cases remain unresolved.^12^

Our first patient was treated with sequestrectomy under general anesthesia, along with antibiotic therapy, and with no further reconstruction. The outcome of the treatment was considered good, and a 3-year follow-up of the patient revealed no evidence of infection. The second patient was referred to an endodontist and maxillofacial surgeon for root canal therapy and extraction of the infected teeth.

## Conclusion

Benign osteopetrosis, is a rare disorder, which might be characterized by an asymptomatic clinical picture. Therefore, a proper clinical and radiographic investigation is essential for accurate diagnosis. Moreover, because of the high infection risk and increased susceptibility to jaw fracture in these patients, careful treatment planning is important to avoid secondary complications of the disease.

## References

[1] Del Fattore A, Peruzzi B, Rucci N, Recchia I, Cappariello A, Longo M (2006). Clinical, genetic, and cellular analysis of forty-nine osteopetrotic patients: Implications for diagnosis and treatment. J Med Genet.

[2] Tolar J, Teitelbaum SL, Orchard PJ (2004). Mechanisms of disease: Osteopetrosis. N Engl J Med.

[3] Krithika C, Neelakandan RS, Sivapathasundaram B, Koteeswaran D, Rajaram PC, Shetkar GS (2009). Osteopetrosis-associated osteomyelitis of the jaws: a report of 4 cases. Oral Surg Oral Med Oral Pathol Oral Radiol Endod.

[4] Ogütcen-Toller M, Tek M, Sener I, Bereket C, Inal S, Ozden B (2010). Intractable Bimaxillary Osteomyelitis in Osteopetrosis: Review of the Literature and Current Therapy. J Oral Maxillofac Surg.

[5] Barry CP, Ryan CD, Stassen LF (2007). Osteomyelitis of the Maxilla Secondary to Osteopetrosis: A Report of 2 Cases in Sisters. J Oral Maxillofac Surg.

[6] Yamada T, Mishima K, Imura H, Ueno T, Matsumura T, Moritani N (2009). Osteomyelitis of the mandible secondary to infantile osteopetrosis: A case report. Oral Surg Oral Med Oral Pathol Oral Radiol Endod.

[7] Xue Y, Wang W, Mao T, Duan X (2012). Report of two Chinese patients suffering from CLCN7-related osteopetrosis and root dysplasia. J Craniomaxillofac Surg.

[8] Junquera L, Rodríguez-Recio C, Villarreal P, García-Consuegra L (2005). Autosomal dominant osteopetrosis and maxillomandibular osteomyelitis. Am J Otolaryngol.

[9] Lin HM, Chang CT, Huang CC (2011). Autosomal dominant osteopetrosis type II. Intern Med.

[10] Cleiren E, Bénichou O, Van Hul E, Gram J, Bollerslev J, Singer FR (2001). Albers-Schönberg disease (autosomal dominant osteopetrosis, type II) results from mutations in the ClCN7 chloride channel gene. Hum Mol Genet.

[11] Bénichou O, Cleiren E, Gram J, Bollerslev J, de Vernejoul MC, Van Hul W (2001). Mapping of Autosomal Dominant Osteopetrosis Type II (Albers- Schönberg disease) to Chromosome 16p13.3. Am J Hum Genet.

[12] Albuquerque MA, Melo ES, Jorge WA, Cavalcanti MG (2006). Osteomyelitis of the mandible associated with autosomal dominant osteopetrosis: A case report. Oral Surg Oral Med Oral Pathol Oral Radiol Endod.

[13] Key LL Jr, Ries WL (2008). Osteopetrosis. In: Bilezikian JP, Raisz LG, Martin TJ, eds. Principles of Bone Biology.

[14] Balemans W, Van Wesenbeeck L, Van Hul W (2005). A Clinical and Molecular Overview of the Human Osteopetroses. Calcif Tissue Int.

[15] Imanimoghaddam M, Davachi B, Nemati S, Johari M (2009). Dental Radiographic Findings of Malignant Osteopetrosis: Report of Four Cases. Iran J Radiol.

[16] Del Fattore A, Cappariello A, Teti A (2008). Genetics, pathogenesis and complications of osteopetrosis. Bone.

[17] Anderson PE, Bollerslev J (1987). Heterogeneity of autosomal dominant osteopetrosis. Radiology.

[18] Frattini A, Vezzoni P, Villa A (2005). The genetics of dominant osteopetrosis. Drug Discov Today Dis Mech.

[19] de Oliveira Hdo C, Pereira Filho VA, Gabrielli MF, Gabrielli MA, Vieira EH (2011). Marginal resection for treatment of mandibular osteomyelitis associated with osteopetrosis: Case report. J Craniomaxillofac Surg.

[20] Vázquez E, López-Arcas JM, Navarro I, Pingarrón L, Cebrián JL (2009). Maxillomandibular osteomyelitis in osteopetrosis. Report of a case and review of the literature. Oral Maxillofac Surg.

